# Functional characterization of vitamin B_12_ from an extremophile—*Pseudomonas alcaliphila* and assessment of its microbial chassis potential

**DOI:** 10.3389/fmicb.2025.1654548

**Published:** 2025-10-03

**Authors:** Sathya Narayanan Venkatesan, Mugesh Sankaranarayanan, Karthik Loganathan

**Affiliations:** ^1^Department of Biotechnology, Vel Tech Rangarajan Dr. Sagunthala R&D Institute of Science and Technology, Chennai, Tamil Nadu, India; ^2^Park’s Biolabs LLP, Chennai, Tamil Nadu, India; ^3^Arqgene, Vellore, Tamil Nadu, India

**Keywords:** cobalamin, *Pseudomonas alcaliphila*, bioactivity, 3-hydroxypropionic acid, *in vitro*, *in silico*, *in vivo*

## Abstract

**Introduction:**

Vitamin B_12_ (B_12_) is an essential cofactor for key metabolic processes in most living organisms, yet only certain bacteria can synthesize it *de novo*. Common forms of B_12_ include adenosylcobalamin (AdoCbl), methylcobalamin (MeCbl) and cyanocobalamin (CNCbl). This study presents the B_12_ production capability of an extremophile—*Ectopseudomonas alcaliphila* MSJ19, and a multilevel evaluation of bioactivity of various B_12_ forms.

**Methods:**

B_12_ extracted from *Ectopseudomonas alcaliphila* MSJ19 was initially analyzed by bioassay and LC–MS to confirm the presence of natural B_12_ forms, followed by *in vitro* enzyme activity assays with glycerol dehydratase (GD) and diol dehydratase (DD). The functionality of various B_12_ forms on these enzymes was further evaluated using in-silico molecular docking studies. The bioactivity at the *in vivo* level was assessed by introducing a coenzyme B_12_-dependent 3-hydroxypropionic acid (3-HP) biosynthetic pathway in *E. coli* W and *Ectopseudomonas alcaliphila* MSJ19 for their ability to transform glycerol into 3-HP.

**Results:**

Bioassay and LC–MS analysis confirmed the presence of ~7 μg/g cdw B_12_ in the processed extract and specific precursor-product ion transitions, indicated the production of natural B_12_ forms. To functionally validate the bioactivity of the crude B_12_ extract, the coenzyme B_12_-dependent 3-HP biosynthesis pathway was employed in recombinant *E. coli* W. Supplementation with different B_12_ forms revealed a hierarchical GD and DD activity (AdoCbl > MeCbl > CNCbl) and a dose-dependent increase in 3-HP production, with an optimal threshold around 500 nM. The conformational specificity of AdoCbl and competitive inhibition of CNCbl and MeCbl were supported by molecular docking of all 3 B_12_ forms with GD and DD. Notably, crude B_12_ extract at 0.35 nM yielded 5.9 mM 3-HP titer, closely matching the 7.8 mM obtained with AdoCbl, confirming its bioactive equivalence. Furthermore, recombinant *Ectopseudomonas alcaliphila* MSJ19 (*Ea*M_r_) harboring the 3-HP pathway produced up to 3.3 mM 3-HP without external B_12_ supplementation, highlighting innate capability of the host to produce and utilize bioactive B_12_*in vivo*.

**Discussion:**

Collectively—*in vitro*, in silico and in vivo approaches establish a functional framework for certifying B_12_ bioactivity and demonstrating *Ea*M as a potent chassis for production of value-added chemicals.

## 1 Introduction

Vitamin B_12_ (B_12_) is a unique cobalt-containing tetrapyrrole cofactor essential for diverse metabolic processes in prokaryotes and eukaryotes (Spataru, 2024). Clinically, B_12_ holds significant importance, as its deficiency is prevalent among all age groups and linked to pernicious anemia and several neurological diseases (Niklewicz et al., 2023). Despite its critical role in most living organisms, only bacteria are capable of synthesizing it *de novo* in two biologically active forms, such as adenosylcobalamin (AdoCbl) and methylcobalamin (MeCbl). Due to very low thermostability and high photosensitivity, these natural forms are often chemically modified into a stable cyanocobalamin (CNCbl) form. Hydroxocobalamin is another commonly found B_12_ form; however, its applications aren’t widespread compared to others. Among these, MeCbl acts as a cofactor only for methionine synthase (MS) in mammals and bacteria. AdoCbl, on the other hand, supports a broad range of coenzyme B_12_-dependent enzymes known as isomerases, which include mutases, eliminases, and amino mutases. Methyl malonyl-CoA mutase (MMUC) is a well-recognized coenzyme B_12_-dependent enzyme in humans, while other enzymes have been identified in bacteria, including *β*-lysine-5,6-aminomutase (LAM), 2-methylene glutarate mutase (MGM), diol dehydratase (DD), D-ornithine-4,5-aminomutase (OAM), ethanolamine ammonia lyase (EAL), glutamate mutase (GM), glycerol dehydratase (GD), isobutyryl-CoA mutase (IM), and ribonucleoside triphosphate reductase (RTPR) (Montoya and Escobar-Briones, 2025). Several of these have been characterized well, in which two isofunctional enzymes—glycerol dehydratase and diol dehydratase are of particular interest in this study, due to their role beyond bacterial metabolism, as catalysts for platform chemical production such as 1,3-propanediol, 3-hydroxypropionic acid, 1-propanol and butanone (Madavi et al., 2024; Brown, 2005). Generally, coenzyme B_12_-dependent enzymes catalyze intramolecular 1,2—rearrangements mediated through the 5′-deoxyadenosyl radical of coenzyme. Substrate binding to the enzymes generates the active radical by homolytic cleavage of the Co-C bond. This highlights the role of active B_12_ forms in mediating such radical-based catalysis (Brunold, 2005).

Over the years, a wide range of B_12_ quantification and characterization methods have been developed including: Microbiological assay, High-performance liquid chromatography (HPLC)—Diode Array Detector (DAD), Liquid chromatography—Mass Spectrometer (LC–MS), UV–vis spectrometry, Raman scattering, atomic absorption spectrometry, Immunoassay, Fluorescence detection, chemiluminescence, capillary electrophoresis, surface plasmon resonance and induced coupled plasma-MS (ICP-MS) (Yang et al., 2024; Trad et al., 2025; Guo et al., 2024; Kansay et al., 2024; Fan et al., 2025). These techniques have been instrumental in analyzing B_12_ from various samples such as pharmaceutical, nutraceutical, and food products, bacterial cultures, serum, seaweeds, algae and mushrooms. Common challenges encountered in B_12_ quantification are low B_12_ concentration in samples often below the limit of detection (LOD) of many methods, stability and sensitivity factors, sample matrix interference, complexity of extraction, sample pretreatment and analytical procedures, and co-detection of B_12_ analogs like cobinamide, cobamide, cobyric acid and pseudo-B_12_ (Santos et al., 2024; Lu et al., 2025; Konings et al., 2024; Deptula et al., 2017). B_12_ analogs are majorly found in bacterial fermentation extracts, hence sample pretreatment steps like solid phase extraction (SPE) and immunoaffinity purification, along with LC–MS, were beneficial in distinguishing bioactive B_12_ forms from B_12_ analogs. Though chromatographic methods can distinguish and quantify active B_12_ forms, they offer little insight into the biological functionality of the B_12_ present (Xie et al., 2019; Chamlagain et al., 2024; Koseki et al., 2023). In contrast, bioactivity assay of B_12_ extracts can be obtained only through the measurement of biological output such as cell growth, protein expression, enzyme activity and biochemical production. Conventional microbiological assay using *Lactobacillus leichmannii* and auxotrophic mutants of *Salmonella typhimurium*, and *Escherichia coli* serve as perfect examples for both quantification and bioactivity evaluation of B_12_ (Raux et al., 1996; Bhushan et al., 2016). In addition, recent developments on PCR-based strategies provide confirmation for B_12_ production on a genotypic level (Venkatesan et al., 2024). Yet they fail to distinguish various forms of B_12_ and are prone to false positives by B_12_ analogs and sample matrix, thus requiring extensive sample pretreatment (Kong et al., 2017; Li et al., 2017). Each B_12_ quantification method has its pros and cons; most importantly, this study does not aim to replace or challenge well-established B_12_ analytical methods. Rather, it focuses on the lacuna in evaluating the bioactivity of various forms of B_12_ from a natural producer in terms of functional biological output.

This work aims to analyze the bioactivity of crude B_12_ extracted from a novel extremophilic B_12_ producer. Through confirming the production of natural B_12_ forms by *Ectopseudomonas alcaliphila* MSJ19 (*Ea*M), the study navigates toward *in vitro*, in silico and *in vivo* approaches to evaluate B_12_ bioactivity and shed light on the effect of various B_12_ forms on bioactivity. The outcomes provide valuable insights into the functionalities of B_12_ from natural producers and the significance of B_12_ dose and forms in clinical and industrial applications. The developed framework to functionally characterize B_12_ is intended to trigger more research focus toward the development of high-throughput biological output-based B_12_ quantification. Finally, the host’s capability to produce biologically active B_12_ has been channeled toward 3-hydroxypropionic acid production in *E. coli* W and *Ea*M by metabolic engineering approaches. *Ectopseudomonas alcaliphila* MSJ19 is an extremophile with psychrophilic (growth at 4–40 °C) and alkaliphilic (optimal pH 9–10) properties. To our knowledge, this represents the first report evaluating B_12_ bioactivity from an extremophilic strain (Venkatesan et al., 2024; Yumoto et al., 2001). The alkaliphilic nature provides revolutionary bioprocess advantages such as pH-based bio-containment that prevents mesophilic contamination, elimination of complex buffering systems and potential compatibility with non-sterile fermentation infrastructure (Zeng et al., 2023; Wernick et al., 2016). Thus, providing a scope for *Ectopseudomonas alcaliphila* MSJ19 as a potent microbial chassis for the sustainable production of value-added biochemicals.

## 2 Materials and methods

### 2.1 Chemicals, strains, and plasmids

All chemicals, reagents and media were correspondingly purchased from SRL-India, Sigma Aldrich, TCI chemicals and Himedia. Yeast alcohol dehydrogenase (*yADH*) was purchased from Sigma-Aldrich. *Ectopseudomonas alcaliphila* MSJ19 was isolated in our previous study, and its 16S rRNA sequence has been deposited in GenBank (ID: PX397011). Plasmid pDK7 (p15a)/*pddCDE*, *gdrAB* was developed by amplification of *pddCDE* genes from genomic DNA isolated from *Klebsiella pneumoniae* 109 and subsequently cloned into *Kpn*I and *Hind*III restriction sites of pDK7 (p15a)/*dhaB123*, *gdrAB* plasmid. The plasmids were transformed into appropriate hosts following the protocol adopted from (Zhou et al., 2013). All strains, plasmids and primers used in this study are listed in Table 1.

**Table 1:** List of bacterial strains and plasmids used in this study.

Strains and plasmids	Description	Source
Strains		
*E. coli* W	Wild-type strain	Sankaranarayanan et al. (2014)
*E. coli* DH5α	Cloning host	MTCC, India
*Klebsiella pneumoniae* MTCC 109	Source for *pddCDE* gene encoding for diol dehydratase	MTCC, India
*Ec*W GD	Recombinant *E. coli* W harboring pUC19/*KGSADH* (Aldehyde dehydrogenase) and pDK7 (p15a)/*dhaB123* (Glycerol dehydratase)*, gdrAB* (Glycerol dehydratase reactivation factors)	Sankaranarayanan et al. (2017)
*Ec*W DD	Recombinant *E. coli* W harboring pUC19/*KGSADH* and pDK7 (p15a)/*pddCDE, gdrAB*	This study
*Ectopseudomonas alcaliphila* MSJ19	An extremophilic B_12_ producer isolated from marine sources in our previous study	Venkatesan et al. (2024)
*Ea*M_r_	Recombinant *Ectopseudomonas alcaliphila* MSJ19 harboring pUCPK/ *dhaB123, gdrAB*, *KGSADH*	This study
*Salmonella typhimurium* Δ*metE* Δ*cbiB*	Strain used for B_12_ bioassay	Thi Nguyen et al. (2021)
Plasmids		
pDK7 (p15a)/*dhaB123*, *gdrAB*	*dhaB123, gdrAB* in pDK7 plasmid; Cm^r^	Ashok et al. (2013)
pDK7 (p15a)/*pddCDE, gdrAB*	*pddCDE*, *gdrAB* in pDK7 plasmid; Cm^r^	This study
pUC19/*KGSADH*	*KGSADH* in pUC19; Km^r^	Ravi and Sankaranarayanan (2023)
pUCPK/*dhaB123, gdrAB*, *KGSADH*	*dhaB123, gdrAB,* and mutant *KGSADH* in pUCPK; Km^r^	Thi Nguyen et al. (2021)
Primers (Forward—F; Reverse—R)	Sequence (5′–3′)	Restriction enzymes
*pddC* F	CGGGTACCATGAGATCGAAAAGATT	*Kpn*I
*pddE* R	GTCAAGCTTTTAATCGTCGCCTT	*Hind*III

Underlined sequences in Primer indicate the incorporated restriction enzyme recognition site.

### 2.2 Shake flask production of vitamin B_12_ by *Ectopseudomonas alcaliphila* MSJ19

Overnight lysogeny broth (LB) *Ea*M culture was pre-cultured in LB medium until mid-late log phase of growth. Subsequently, 0.1 OD_600_ of exponentially grown cells was reinoculated appropriately in LB production medium containing precursors: CoCl_2_ (5 mg/L), DMBI (75 mg/L), and Betaine (1 g/L) and incubated under aerobic conditions at 37 °C, 200 rpm. Wild-type *E. coli* W were cultivated under similar conditions to serve as a negative control wherever appropriate in this study. Cell growth was measured at regular intervals by a UV–Vis spectrophotometer, and after 18 h, cells were harvested for B_12_ extraction (4,500 rpm, 15 min). One OD_600_ corresponds to 0.33 g (±0.05 g) of dried cell mass per liter (Arasu et al., 2013).

### 2.3 Extraction and quantification of B_12_

Cells were washed twice with 100 mM potassium phosphate buffer (pH 7.0) and resuspended in the same buffer for B_12_ extraction under ice with minimal light exposure. Cell concentration was measured before and after lysis. Cells were lysed by ultrasonication at 30% amplitude for 6 min with a 10-s ON/OFF cycle (VCX 130, Sonics; 20 kHz), centrifuged (4,500 rpm, 10 min), supernatant filtered through a 0.22 μm syringe filter and used as crude B_12_ extract for further analysis.

For B_12_ quantification by bioassay, the protocol mentioned in our previously published study was followed exactly (Venkatesan et al., 2024). To convert natural B_12_ forms into the more stable CNCbl form, 0.1% w/v NaCN was added to the crude B_12_ extract, and after 5 min incubation (37°C), the mixture was autoclaved (121 °C, 15 min) and cooled on ice. The samples were centrifuged (4,500 rpm, 20 min) and the supernatant was passed through a 0.22 μm syringe filter prior to LC–MS analysis. The LC–MS analysis was performed for both crude B_12_ extract and cyano-converted extract, using a Waters TQD LC–MS/MS system equipped with a Kinetex (2.6 μm, XB C18 Column, 2.1 × 100 mm). 20 mM ammonium formate in water (Mobile Phase A) and methanol (Mobile Phase B) were used for sample elution under the following linear gradient: 90% mobile phase A for 0–2 min, 90% mobile phase A for 2–4 min, 10% mobile phase A for 4–5 min, 90% mobile phase A for 5–7 min. The flow rate was maintained at 0.3 mL/min, the column temperature was set to 35 °C, and the injection volume was 10 μL. Mass spectrometry was conducted in positive electrospray ionization (ESI) mode with a source temperature of 140°C, desolvation temperature of 300 °C, cone gas flow of 10 L/h and desolvation gas flow of 1,000 L/h. The capillary voltage was set at 26 V, and the cone voltage was 35 V. The quantification of CNCbl was performed using multiple reaction monitoring (MRM) transitions, monitoring the precursor ion at m/z 678.5 and the product ions at m/z 147.0 and 358.9, with collision energies of 34 eV and 24 eV, respectively (Stumpf et al., 2024; Kahoun et al., 2022). This targeted MRM setting was chosen to achieve high specificity and sensitivity for CNCbl, while avoiding cross-detection of other B_12_ forms in crude extracts (Reddy KotamReddy et al., 2023). The same LC–MS method was also adopted for crude B_12_ extracts.

### 2.4 Enzyme activity assay

The modified M9 medium used for shake flask studies in *Ec*W GD and *Ec*W DD contained: MgSO_4_·7H_2_O, 0.5 g/L; NaCl, 1.0 g/L; NH_4_Cl, 1.0 g/L; yeast extract, 1 g/L; glycerol, 100 mM; potassium phosphate buffer (pH 7.0), 100 mM; kanamycin 50 mg/L; and chloramphenicol 25 mg/L. Unless stated otherwise, LB medium and the same modified M9 medium with appropriate antibiotics were used for primary inoculum and secondary inoculum, respectively. Shake flask cultivation was carried out with a working volume of 50 mL culture with an inoculum of 0.1 OD_600_ in a 250 mL Erlenmeyer flask at 37°C, 250 rpm under aerobic conditions. For enzyme production, the cultures were induced at 0.6 ± 0.05 OD_600_ with 0.5 mM IPTG. After 6 h incubation, cells were harvested (5,000 rpm, 15 min) and washed once with 20 mM potassium phosphate buffer. Subsequently, cells were resuspended in the same buffer and subjected to ultrasonication under ice at 30% amplitude for 4 min with a 10-s ON/OFF cycle. The obtained lysate was centrifuged (13,000 rpm, 30 min), and the supernatant was collected to measure total protein concentration (by the Bradford method), glycerol dehydratase (GD) and diol dehydratase (DD) activity, respectively.

GD activity was measured by following the protocol developed by Sankaranarayanan et al. (2017), and the same method was employed to measure DD activity. Briefly, the substrate mixture (~1.8 mL), containing 20 mM potassium phosphate buffer (pH 8.0), 3 mM MgCl_2_ and 40 mM 1,2-PDO, was placed in a 1-cm path length spectrophotometer cuvette. B_12_ solution (100 μL) was added to this assay mixture, containing 0.15 mM NADH and 1.5 mM ATP. B_12_ concentration and type were varied individually to study their effects. Then, the coupling enzyme (40 μL) yADH (12 U/mL) was added using an air-tight gas chromatography syringe, and the cuvette was incubated for 3 min in a water bath at 37 °C. The enzymatic reaction was initiated by injecting 50 μL of crude GD or crude DD enzyme solution, appropriately (typically <0.03 U/mL). The NADH concentration was determined at 340 nm with the extinction coefficient (ε_340_) of 6.22 mM^−1^ cm^−1^ on a UV spectrophotometer. One unit of GD or DD activity was defined as the amount of enzyme required to convert 1 μmol of 1,2-PDO to propionaldehyde per minute under given assay conditions.

### 2.5 Molecular docking of various B_12_ forms with GD and DD

Molecular docking was performed between each B_12_ form—AdoCbl, CNCbl, MeCbl and the active sites of GD and DD. High-resolution crystal structures of GD (PDB ID: 1IWP) and DD (PDB ID: 1DIO) were retrieved from the protein data bank (PDB) (Yamanishi et al., 2002; Shibata et al., 1999). The crystal structures were refined by eliminating water molecules and ligands using PyMOL software (version 3.1.3). Refined proteins were subsequently processed using Autodock tools (v 1.5.7) by setting grid parameters for both GD and DD based on reference active site coordinates reported already (Yamanishi et al., 2002; Masuda et al., 2000). 3D structure of all the ligand molecules AdoCbl, CNCbl, and MeCbl were procured from protein structures (PDB ID: 5C8A, 5NP4, 3SC0) co-crystalized with respective ligands. Each B_12_ ligand was assigned its respective charges and docked into the aforementioned active site grid. Docked conformations exhibiting higher binding and similar interactions with key active site residues were considered for further evaluation.

### 2.6 Shake flask 3-HP production in *Ec*W GD and *Ec*W DD

Shake flask 3-HP production with the respective host was carried out aerobically using the same modified M9 medium (50 mL) with a starting inoculum of 0.1 OD_600_ in a 250-ml Erlenmeyer flask incubated at 37°C, 250 rpm. The cultures were induced at 0.6 ± 0.05 OD_600_ with 0.1 mM IPTG and supplemented with various forms and concentrations of B_12,_ respectively, at 3, 6, 9, and 12 h of cultivation. The details on B_12_ form and concentration supplemented for each shake flask experiment were furnished in Supplementary Table 1. Samples were collected periodically to determine the cell mass, residual substrate and metabolites. Briefly, the collected culture samples were centrifuged (10,000 rpm, 10 min), then the supernatant was diluted appropriately and filtered using a 0.22 μm PVDF membrane filter (Millipore). Then the samples were passed through an HPLC system equipped with an Aminex HPX-87H column (300 mm × 7.8 mm, Bio-Rad, United States) maintained at 65°C. The mobile phase consisted of 2.5 mM H_2_SO_4_ with a flow rate of 0.5 mL/min, and metabolite concentrations were analyzed using a Refractive Index Detector (RID) and a Photo-diode array detector (PDA) (Ravi and Sankaranarayanan, 2024).

### 2.7 Shake flask 3-HP production in recombinant *Ectopseudomonas alcaliphila* MSJ19 (*Ea*M_r_)

Shake flask cultivation of the *Ea*M_r_ for 3-HP production was carried out in the same B_12_ production medium with the addition of Kanamycin (30 mg/L). The cultivation was carried out aerobically at 37°C, 200 rpm, with an initial cell concentration of 0.1 OD_600_. 100 mM of glycerol (carbon source for 3-HP production) was added when the cell concentration reached 0.7 ~ 1 OD_600_. The samples were withdrawn periodically to determine the cell mass, glycerol, 3-HP and other metabolites.

## 3 Results

### 3.1 Production of natural forms of B_12_ by *Ectopseudomonas alcaliphila* MSJ19

**Figure 1:**
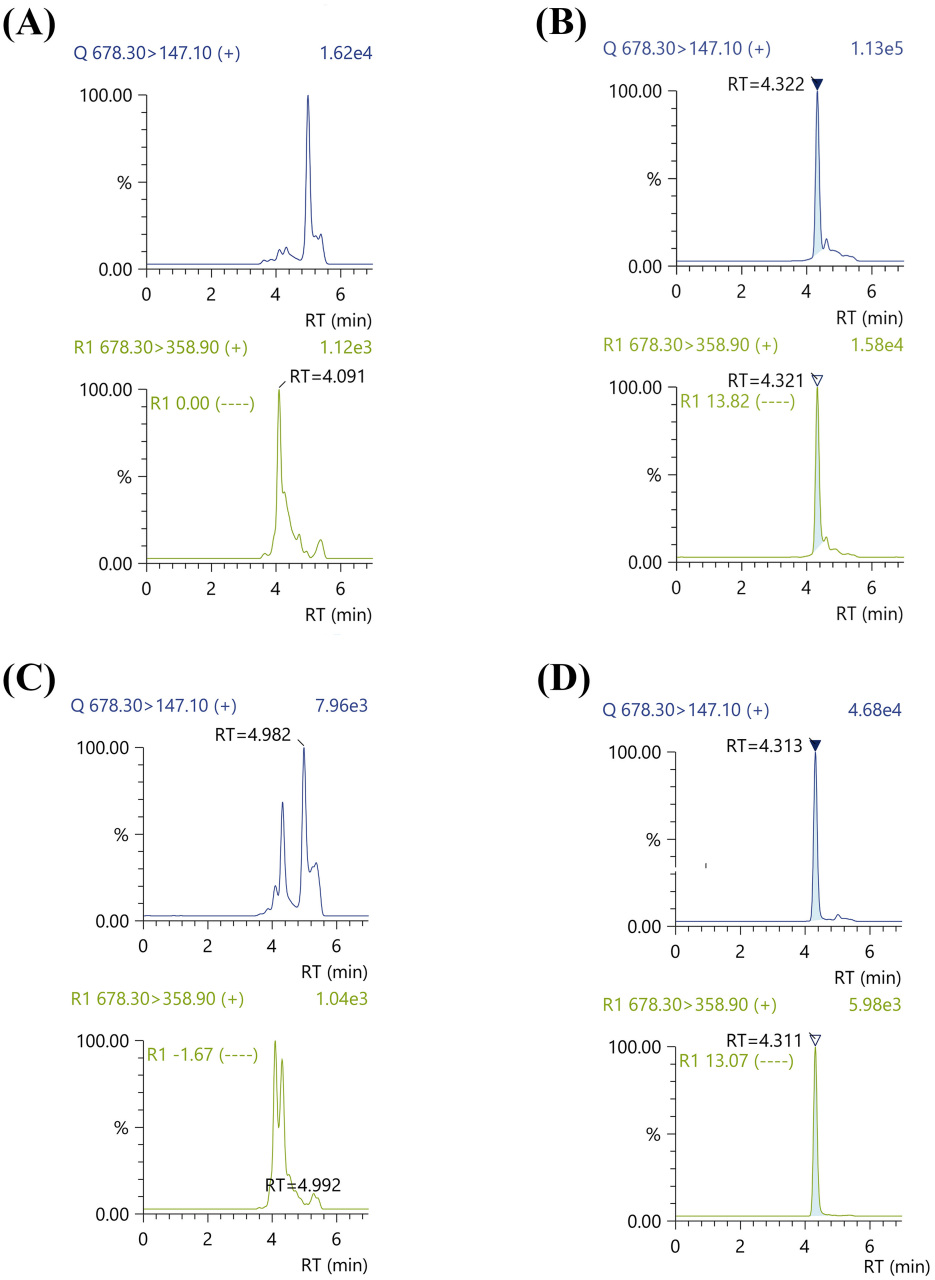
LC–MS/MS chromatograms for confirmation of natural B_12_ forms in *Ectopseudomonas alcaliphila* MSJ19 extract under specific MRM transitions: **(A)** Crude B_12_ extract (no peak observed at RT ~ 4.32 min), **(B)** Cyano-converted B_12_ extract (distinct peak observed at RT 4.32 min, matching CNCbl standard), **(C)** Crude B_12_ extract spiked with 0.5 μM MeCbl and AdoCbl (no peak observed at RT ~ 4.32 min) and **(D)** CNCbl (concentration = 5 ppb; clear peak at RT 4.31 min).

Consistent with our previous study, the B_12_ levels of *Ectopseudomonas alcaliphila* MSJ19 (*Ea*M) quantified by bioassay were 7.18 μg/g cdw (Venkatesan et al., 2024). To validate B_12_ forms, LC–MS analysis was performed for: (a) crude B_12_ extract, (b) cyano-converted B_12_ extract, and (c) crude B_12_ extract spiked with 0.5 μM each of standard AdoCbl and MeCbl (Figure 1). A distinct peak at RT 4.32 min was observed for cyano-converted B_12_ extract corresponding to MRM transitions m/z 147.0 and 358.9, matching precisely with the standard CNCbl profile. These transitions were selected based on their high sensitivity and specificity for CNCbl quantification. The concentration of B_12_ was quantified as 6.93 μg/g cdw. In contrast, no corresponding peaks were observed at RT 4.3 min for either the crude extract or the spiked crude extract, indicating that CNCbl was not natively present in the bacterial extract. These results collectively support that *Ea*M produces only the natural (coenzyme) forms of B_12_—namely, MeCbl and AdoCbl—which are not detected in this LC–MS method due to their distinct transition requirements (such as m/z 685.6 and 665.6, respectively) (Heal et al., 2014). By providing a clear distinction between the presence of natural and non-natural forms of B_12_, the present strategy offers a streamlined and scalable framework for both qualitative and quantitative assessment of B_12_ production in industrially relevant microbial strains.

### 3.2 *In-vitro* bioactivity evaluation of crude B_12_ extract

Glycerol dehydratase (GD) and diol dehydratase (DD) are isofunctional, coenzyme B_12_-dependent enzymes, whose characteristics and *in vitro* assays have been well studied (Toraya et al., 2022; Nasir et al., 2020). These enzymes are known to be catalytically active only in the presence of AdoCbl with varying degrees of sensitivity (Marsh and Meléndez, 2012; Toraya et al., 1979). While other B_12_ forms, such as MeCbl and CNCbl, are often reported as competitive inhibitors (Poppe and Rétey, 1997; Toraya and Ishida, 1991). These features make GD and DD valuable *in vitro* tools for evaluating the functional bioactivity of B_12_ from bacterial extracts.

**Figure 2:**
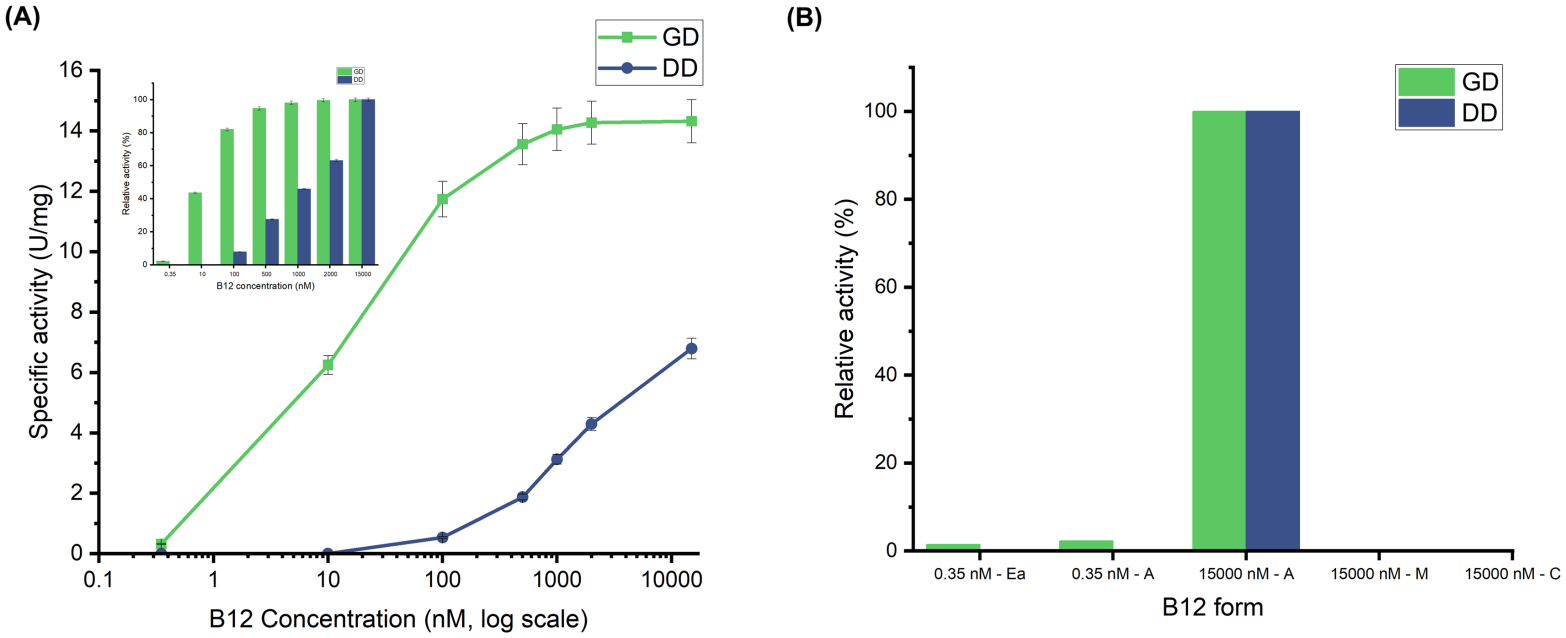
Harnessing *in vitro* enzyme activity assays of GD and DD to evaluate B_12_ bioactivity: **(A)** AdoCbl concentration-dependent variation of GD and DD activity (U/mg)—Concentration (nM) is plotted on a logarithmic scale. The inset shows relative activities (%) of GD and DD normalized to their respective maximum activities. **(B)** Effect of B_12_ forms on GD and DD activity; Experimental groups: *Ea*—*Ectopseudomonas alcaliphila* MSJ19 crude B_12_ extract; A, Adeonsylcobalamin; M, MeCbl; C, CNCbl. Error bars represent standard deviation from three independent biological replicates (*n* = 3).

Generally, activity assays for these isofunctional enzymes are performed at a saturated coenzyme B_12_ concentration of around 10–20 μM (Kumar et al., 2016; Wei et al., 2014). However, due to the low concentration of crude B_12_ used for this study (0.35 nM), a preliminary investigation was carried out to study the effect of AdoCbl concentration on GD and DD activity. Maximal activities were observed at 15 μM AdoCbl, yielding 14.32 U/mg for GD and 6.79 U/mg for DD. At 0.35 nM, the enzyme activity dropped to 0.32 U/mg for GD, while no significant activity was observed for DD (Figure 2A). The difference in activities between GD and DD correlates with their known kinetic parameters, specifically the reported *K_m_* values of GD (~ 8 nM to 20 nM) and DD (~ 0.7 μM) from *Klebsiella* sp. (Yamanishi et al., 2002; Wang et al., 2007). According to previous reports, GD attained 95% of its maximum activity and DD only 4% at 120 nM AdoCbl (Yamada et al., 2004). Relatively, the current study shows that GD and DD attained 81% and 7% of their respective maximum activities at 100 nM AdoCbl, confirming the accuracy of the assay and reinforcing AdoCbl sensitivity among the enzymes. As anticipated, no significant enzyme activity was observed when the assay was performed with CNCbl and MeCbl, even at 15 μM, the saturated concentration used for AdoCbl (Figure 2B). The missing 5′-deoxyadenosyl radical upon binding of CNCbl and MeCbl to the enzyme is expected to be the sole reason for their inability to support GD and DD activity (Toraya, 2000; Bucher et al., 2012). Previous reports support this by showing that MeCbl and CNCbl act as competitive inhibitors for DD (*K_i_* of 0.73 μM and 1.8 μM, respectively) (Toraya et al., 1977) and CNCbl for GD (*K_i_* = 21.6 nM) (Poppe and Rétey, 1997). Notably, with 0.35 nM of crude B_12_ extract, the GD activity measured was 0.21 U/mg, which was slightly lower than 0.32 U/mg obtained with standard AdoCbl at the same concentration. While this suggests that the crude B_12_ extract predominantly contains AdoCbl, it confirms that *Ea*M has dominantly produced AdoCbl; the lower activity could likely be due to the presence of some MeCbl, which may exert competitive inhibition. However, further studies are required to confirm this hypothesis. These results collectively demonstrate that the B_12_ produced by *Ectopseudomonas alcaliphila* MSJ19 is functionally bioactive, with *in vitro*-based assays providing indirect but reliable confirmation of AdoCbl as the dominant form in the crude extract.

### 3.3 *In silico* prediction of various B_12_ forms reactive specificity with GD and DD

To complement the differential catalytic activity of B_12_ forms on a structural basis, molecular docking was performed between three B_12_ ligands—AdoCbl, CNCbl, and MeCbl—and the known crystal structures of GD and DD. A total of six docking combinations were generated, and a complete summary of interactions and H-bond distances for each docking conformation is provided in Supplementary Table 2. Based on previous crystallographic studies, 12 key active site residues were defined for GD (Yamanishi et al., 2002) and 7 for DD (Masuda et al., 2000) to assess the binding capability of ligands within the functionally active site.

**Figure 3:**
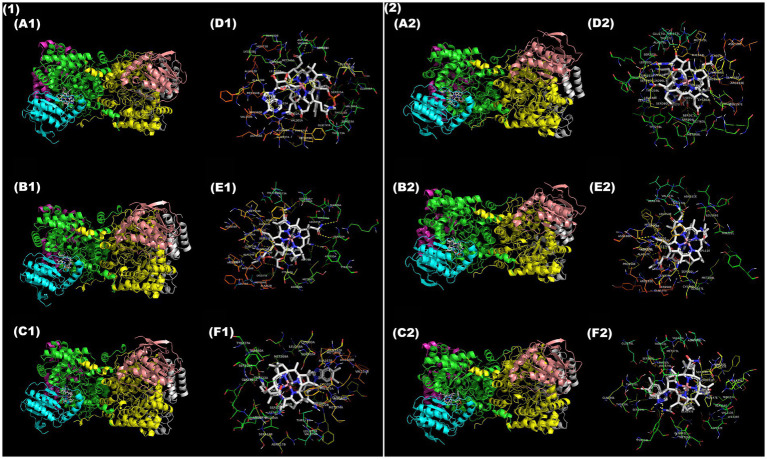
Molecular docking of B_12_ ligands with GD **(1)** and DD **(2)**. Stereo views showing overall GD **(A1–C1)** and DD **(A2–C2)** structure in complex with **(A1,A2)** AdoCbl, **(B1,B2)** CNCbl, and **(C1,C2)** MeCbl. Chains of the GD heterotrimer are colored as follows: A—green, B—cyan, C—magenta, D—yellow, E—salmon, F—grey; Chains of the DD heterotrimer are colored as follows: A—yellow, B—salmon, E—cyan, G—grey, L—green, M—magenta. Zoomed-in interaction maps of ligands with active site residues of GD (D1—F1) and DD (D2—F2): (D1, D2) AdoCbl, (E1, E2) CNCbl, and (F1, F2) MeCbl. Hydrogen bonds and polar interactions are visualized between ligand atoms and neighboring amino acid residues. Ligand atom color scheme: C—grey, N—Navy blue, O—red, S—Orange, Co—pink.

In GD, AdoCbl exhibited the most favorable binding conformation for catalytic function, forming hydrogen bonds with five active site residues (SER122, THR104, SER225, THR173, LYS102) and a binding energy of −3.93 kcal/mol (Figures 3A1,D1). These interactions span both the corrin ring and adenosyl moiety, positioning the ligand in a favorable conformation for Co-C homolysis and radical exchange. CNCbl exhibited a significantly lower binding energy (−14.5 kcal/mol) and formed four interactions with active site residues (SER122, ASP235, ALA124, THR173) (Figures 3B1,E1). While such tight binding reflects higher affinity, the absence of the adenine moiety blocks its catalytic ability and complements its role as a competitive inhibitor. MeCbl also interacted only with one active residue (SER122) and an intermediate binding energy (−5.81 kcal/mol) (Figures 3C1,F1), further reflecting its non-catalytic but potentially competitive inhibitory role.

In DD, AdoCbl again exhibited the highest number of active site interactions, yet fewer than in GD. Only two out of six interactions matched with key active side residues (THR172, SER301) following an intermediate binding energy (−7.23 kcal/mol) (Figures 3A2,D2). This observation aligns with the *in vitro* enzyme activity assay, where DD activity expressed a higher *Km* than GD, thus justifying the lower specificity of AdoCbl with DD. CNCbl had only one matching residue (THR172) among five interactions with a lower binding energy (−12.9 kcal/mol) (Figures 3B2,E2). MeCbl had only one matching residue (SER224) among its two interactions with a higher binding energy (−6.99 kcal/mol) among all 3 B_12_ forms with DD (Figures 3C2,F2). These binding predictions reflect the inferiority of B_12_-driven catalysis with DD as compared to GD.

Importantly, these findings elucidate the conserved structural and functional preference of GD and DD for AdoCbl. Evidently, comparison of available crystal structures and docking combinations of this study has shown that CNCbl and MeCbl are also capable of binding within the active site, but they lack the adenine moiety necessary to trigger Co-C bond homolysis and substrate rearrangements (Shibata et al., 2018). Hence, the adenine moiety not only acts as a radical initiator, but also participates in key interactions to position the cofactor in an appropriate spatial conformation for catalytic activity. Therefore, the *in-silico* findings support the *in vitro* enzymatic assay, confirming that only AdoCbl positions itself in a catalytically active conformation in both GD and DD in a conserved manner. Meanwhile, CNCbl and MeCbl are capable of competitive inhibition due to their catalytically inactive binding conformation.

### 3.4 *In-vivo* bioactivity evaluation of crude B_12_ extract

In the two-step catalytic pathway for 3-HP production, Coenzyme B_12_ (AdoCbl) serves as an essential cofactor for glycerol dehydratase (Kumar et al., 2012). Therefore, 3-HP production can act as a reliable qualitative metric for assessment of B_12_ bioactivity, offering a more meaningful output than conventional microbiological assay. To evaluate this, *Ec*W GD and *Ec*W DD were supplemented individually with AdoCbl, MeCbl, and CNCbl for 3-HP production. Among these, AdoCbl yielded the highest 3-HP production in *Ec*W GD, confirming it as the most effective cofactor for GD activity. Whereas MeCbl resulted in only 63% of this maximum, and CNCbl only 36%. A similar trend was observed for *Ec*W DD, yet its maximum 3-HP titre was only 52% of that achieved with *Ec*W GD. Such a low 3-HP titre of DD in this expression system is obvious due to the following well-documented reasons: (i) 1,2-PDO is the preferred substrate for DD over glycerol (Sauvageot et al., 2002), (ii) absence of diol dehydratase reactivase in this expression system, making DD prone to suicide inactivation like GD in the presence of glycerol (Bilić et al., 2019), (iii) *gdrAB* is known to be ineffective in reactivating DD (Kajiura et al., 2007), and (iv) less B_12_ specificity of DD as observed in enzyme activity analysis (Poppe and Rétey, 1997). These results further demonstrate a substantial decline in GD and DD activity with synthetic B_12_ forms and justify the functional superiority of the natural B_12_ forms.

Notably, the modest 3-HP production with CNCbl suggests that *E. coli* may possess intrinsic metabolic mechanisms to convert CNCbl into biologically active forms, analogous to human metabolic pathways (Kelly, 1997). However, the relatively low 3-HP titer (70% lower than AdoCbl) indicates that this intracellular conversion is likely rate-limiting. The difference in 3-HP production between MeCbl and CNCbl also reflects the metabolic complexity of their respective conversion process, as CNCbl conversion is mediated by a four-step enzymatic process, while MeCbl requires only a single step (Rizzo et al., 2016).

**Figure 4:**
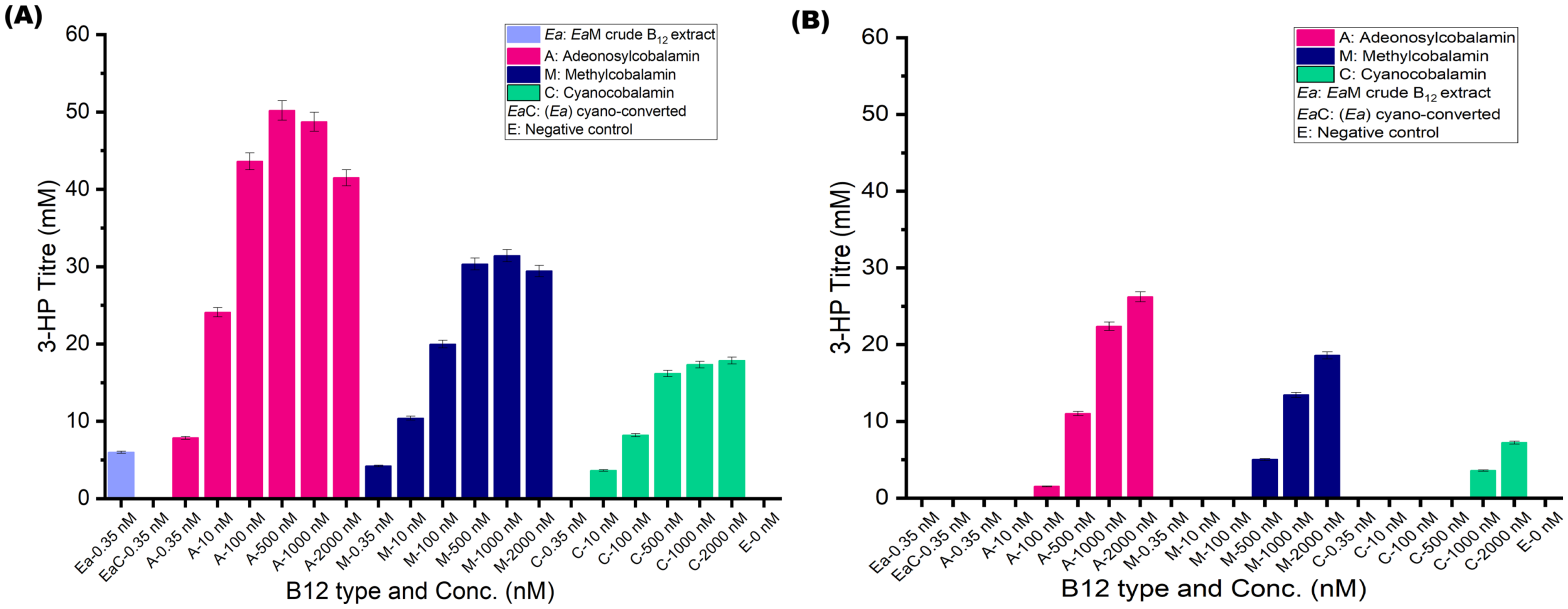
Functional evaluation of B_12_ forms and concentration on 3-HP production by *Ec*W GD and *Ec*W DD, respectively. **(A)** Summary of 3-HP production titre (mM) of *Ec*W GD under supplementation with different B_12_ forms at varying concentrations (nM). **(B)** Summary of 3-HP production titre (mM) of *Ec*W DD under supplementation with different B_12_ forms at varying concentrations (nM). Error bars represent standard deviation from three independent biological replicates.

While earlier studies typically employed 2000 nM AdoCbl for optimal 3-HP production in *E. coli* (Nguyen-Vo et al., 2019), the concentration of crude B_12_ extract used is comparatively less (0.35 nM). Therefore, this study also evaluated the effect of B_12_ concentration on 3-HP production across a wide range (0.35–2,000 nM) for each B_12_ form (Figures 4A,B). Interestingly, B_12_ concentration had a significant effect on 3-HP production, similar to the enzyme activity. Remarkably, the highest 3-HP titer of 50.2 mM was observed at 500 nM AdoCbl for *Ec*W GD, beyond which no significant increase in titre could be observed. The optimal 3-HP production at 500 nM reflects seamlessly with the V_max_ of GD, embarking on the critical impact of B_12_ on the rate-limiting step of the 3-HP catalytic pathway. The 3-HP production titer (41.5 mM) with 2 μM AdoCbl was also consistent with previous reports (Sankaranarayanan et al., 2017). Similar trends were observed for other B_12_ forms. Owing to the higher *K_m_* of DD, 3-HP production was maximal (26.2 mM) only at 2,000 nM and suggesting that further increase in B_12_ may still enhance activity (typically close to the V_max_ of DD (>7 μM)).

Of particular interest, crude B_12_ extract at 0.35 nM supported a 3-HP titre of 5.9 mM in *Ec*W GD—closely matching the 7.8 mM titer at 0.35 nM AdoCbl. This confirms the presence of active B_12_ forms in the extract. The marginal difference could be attributed to the presence of some MeCbl in the extract, as only the total B_12_ concentration was quantified. Consistently, MeCbl at 0.35 nM attained a lower 3-HP titer of 4.2 mM. In contrast, no measurable 3-HP production was observed at 0.35 nM cyano-converted extract, despite a very low 3-HP titer of 1.1 mM at 0.35 nM CNCbl. This suggests potential interference from matrix effects during conversion (Nakos et al., 2017) or simply the titre falling to the limit of detection (LOD = 0.8–1.0 mM). As expected, no 3-HP production was observed in the negative control with *E. coli* W extract, thus justifying that any potential impurities in crude bacterial extracts do not affect 3-HP production. These findings reinforce the presence of a biologically active form of B_12_ in crude extract, and the chemical conversion process has led to a non-natural/synthetic B_12_ form, which obviously has led to a decrease in or no 3-HP production. Collectively, these results strongly establish the utility of 3-HP production as an *in vivo* functional assay for B_12_ bioactivity. Building on these findings, the next section validates the *in vivo* B_12_ bioactivity using recombinant *Ectopseudomonas alcaliphila* MSJ19 itself.

### 3.5 Assessment of the 3-HP production capability of *Ea*M_r_

**Figure 5:**
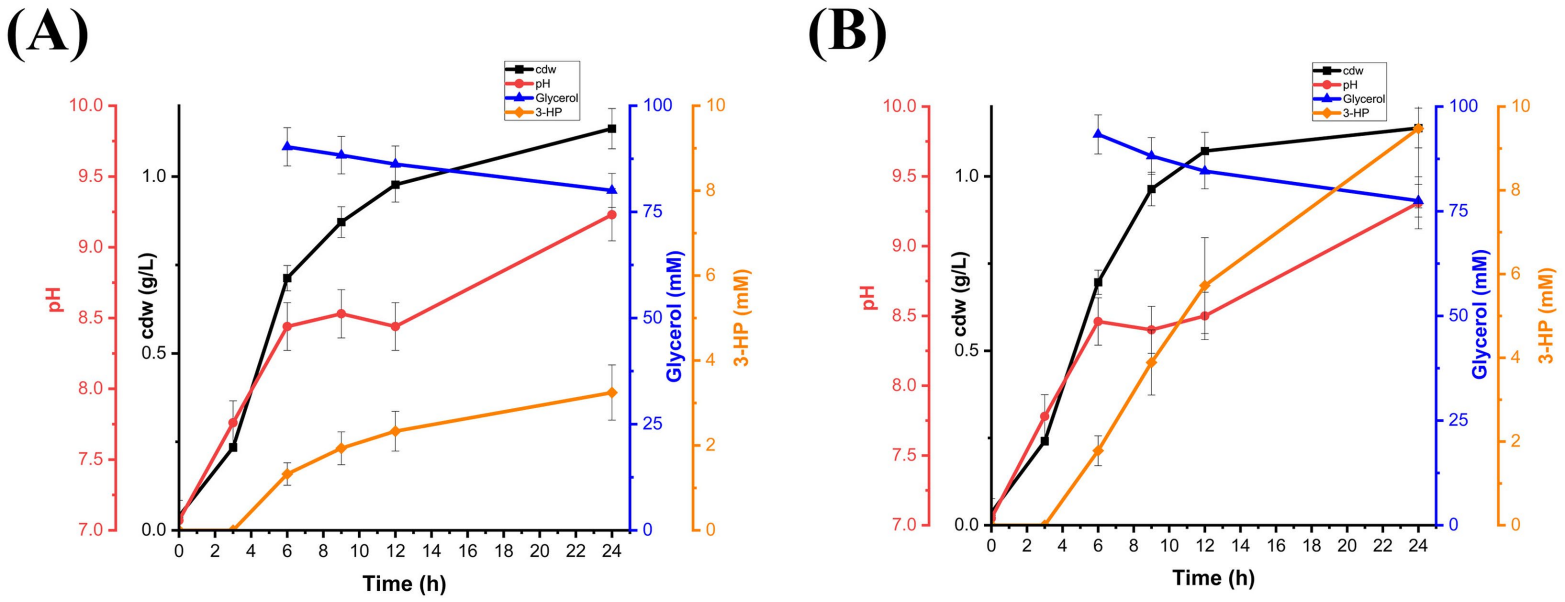
Time-course profile of *Ea*M_r_ showing cell growth (cdw g/L), pH variation, glycerol consumption (mM), and 3-HP production (mM): **(A)** without exogenous AdoCbl supplementation and **(B)** with supplementation of 2 μM AdoCbl. Error bars represent standard deviation from three independent biological replicates.

To evaluate the *in vivo* bioactivity of endogenously produced B_12_, *Ectopseudomonas alcaliphila* MSJ19 was engineered to express the 3-HP biosynthetic pathway via plasmid pUCPK harboring *dhaB123*, *gdrAB*, and *KGSADH*. Shake flask cultivation was performed with and without an exogenous supply of 2 μM AdoCbl, thereby ensuring that any observed 3-HP production is solely dependent on the host’s innate B_12_ biosynthesis capability. Correspondingly, *Ea*M_r_ produced a maximum 3-HP titre of 3.2 mM without external B_12_, indicating the endogenous production of coenzyme B_12_ was sufficient to activate GD and enable 3-HP biosynthesis (Figure 5A). As expected, no 3-HP production was observed in control flasks without glycerol supplementation (data not shown), confirming that 3-HP originated exclusively from glycerol metabolism and not from medium components or endogenous carbon sources. 3-HP production was improved (9.5 mM) under B_12_ supplementation, indicating that 3-HP flux can be further enhanced through B_12_ supplementation (Figure 5B). In addition, glycerol consumption and 3-HP production were relatively low in either case compared to *EcW* GD. This could be presumed due to intrinsic regulatory barriers, such as the presence of transcriptional repressors in the host’s native glycerol catabolic pathway and/or limited compatibility between the heterologous plasmid system and the host transcriptional or translational machinery (Thi Nguyen et al., 2021; Prieto-de Lima et al., 2024). Although elucidating these factors was beyond the scope of this study, the results clearly establish the functional bioavailability of naturally synthesized B_12_ in *Ea*M_r_. These findings not only validate *Ectopseudomonas alcaliphila* MSJ19 as a biologically competent B_12_ producer but also highlight its potential as a versatile microbial chassis for value-added chemical production beyond vitamin B_12_.

## 4 Discussion

Vitamin B_12_ is structurally complex and exists in several natural and synthetic forms. Among them, only AdoCbl and MeCbl are biologically active, serving as cofactors in radical-based and methyl-transfer enzymatic reactions, respectively. Bacteria are the sole workhorses for industrial scale production of this essential vitamin; however, they can produce inactive B_12_ analogs (Thirupathaiah et al., 2012). Therefore, assessing the bioactivity of B_12_ rather than relying only on total B_12_ quantification is essential to grade its functional bioavailability. Recent advancements in chromatographic and immunoassay methods have played a significant role in classifying the forms of B_12_ (Möller et al., 2022; Balabanova et al., 2022). However, studies are limited in evaluating the activity of crude extracts of natural B_12_ producers using a valid biological output (Chamlagain et al., 2021). This study details a biologically integrated workflow combining *in vitro*, *in silico* and *in vivo* approaches to uncover the potential of active forms of B_12_ produced by a novel extremophilic strain (*Ea*M).

Initially, conventional bioassay using *Salmonella typhimurium* Δ*metE* Δ*cbiB* and LC–MS were valuable in confirming the production of natural form (AdoCbl & MeCbl) of B_12_ (~7 μg/g cdw) by *Ectopseudomonas alcaliphila* MSJ19. The establishment of enzymatic assay methods for coenzyme B_12_-dependent enzymes such as GD and DD paved a plausible approach to further study the bioactivity of crude B_12_ extract. The coupled enzymatic method to measure GD activity also stood reliable for DD activity measurement, particularly due to its increased substrate preference to 1,2-PDO (Toraya et al., 2022). Substrate binding to the holoenzyme triggers Co-C bond homolysis, leading to the formation of cob(II)alamin and 5′-deoxyadenosyl radical. Theoretically, this radical is essential to mediate 1,2-rearrangements in the substrate during enzyme catalysis (Giedyk et al., 2015). Justifiable to this, both GD and DD were capable of product formation only in the presence of AdoCbl, while no notable enzyme activity was observed for CNCbl and MeCbl even at very high concentrations (15 μM) due to their inability to form an adenosyl radical.

Interestingly, GD activity with 0.35 nM crude extract was nearly equivalent to that of standard AdoCbl, confirming the dominant presence of AdoCbl in the crude extract. The lack of DD activity with crude extract is attributed to its higher *K_m_* of ~0.8 μM for AdoCbl, further validating the reliability of such enzyme activity assays to confirm B_12_ bioactivity. Despite the non-catalytic activity of other B_12_ forms, they play a larger role as competitive inhibitors, and it is to be realized that their presence in sample extracts tends to underestimate the bioactivity of actual AdoCbl present. The potential inhibitory effects of other B_12_ forms were supported by molecular docking, which revealed comparable binding energies across all B_12_ forms, suggesting competitive inhibition. Thus, *in vitro* assays combined with *in silico* insights reinforce the fact that B_12_ bioactivity is not defined by binding affinity alone, but also by the ability to support 5′-deoxyadenosyl radical generation and substrate rearrangements.

Transitioning toward the applicability of the coenzyme B_12_-dependent 3-HP production pathway in recombinant *E. coli* as an *in vivo* model system for B_12_ bioactivity enlightened the fate of other B_12_ forms beyond competitive inhibition. Contrarily, 3-HP production in recombinant *E. coli* was observed under supplementation of all 3 B_12_ forms individually with varying degrees (AdoCbl > MeCbl > CNCbl). Although in vitro enzyme assay and in silico models have strongly backed the competitive nature of other B_12_ forms on GD and DD, this discrepancy likely arises from the host’s intracellular B_12_ salvage and conversion mechanisms, enabling conversion of other B_12_ forms into AdoCbl. Haptocorrin-based B_12_ binding, absorption by intrinsic factors, innate mechanisms to convert various B_12_ forms into a metabolically active form and bioavailability were well documented in humans (Vincenti et al., 2021). While such B_12_ conversion mechanisms were very scarcely reported in bacterial systems (Reynolds et al., 1980), this study is the first of its kind to report their impact on coenzyme B_12_-dependent platform chemical synthesis. Future work should investigate the regulation of these conversion mechanisms and their fine-tuning to improve the flux of AdoCbl for platform chemical production.

A concentration-dependent variation in 3-HP titre across B_12_ forms, paralleled GD and DD enzymatic activity trends. These outcomes not only validate the presence of natural B_12_ form in *Ectopseudomonas alcaliphila* MSJ19 extract but also demonstrate that AdoCbl is indispensable for GD/DD—mediated bioconversion. Furthermore, the distinct functional differences between natural and synthetic B_12_ forms, along with the concentration thresholds observed, provide the groundwork for future studies to develop a quantitative enzyme-based assay for B_12_. These insights also offer a valuable framework for optimizing 3-HP production.

Finally, recombinant expression of the 3-HP pathway in *Ectopseudomonas alcaliphila* MSJ19 confirms that the host’s endogenously synthesized B_12_ is not only biologically active but also sufficient to support product formation without external B_12_ supply. This approach effectively bypasses the tedious processes for B_12_ extraction, purification and quantification, which often suffer from factors like sample instability, interference from analogs, and the need for advanced instrumentation (Avrămia et al., 2024; Pakeeza et al., 2024). To our knowledge, this is the first instance to report 3-HP production in an extremophilic *Ectopseudomonas* strain without an exogenous supply of B_12_. This acts as a beacon for future avenues to improve B_12_ production in this host and explore its possibilities as a reliable and efficient non-model microbial chassis for other value-added chemicals production.

## 5 Conclusion

This study presented a comprehensive multifaceted approach to evaluate the bioactivity of B_12_ from a natural producer. *In vitro* and in silico investigations have shed light on the specificity of GD and DD toward AdoCbl and revealed the competitive inhibitory effects of other B_12_ forms, likely due to the absence of the essential 5′-deoxyadenosyl radical. An *in vivo* approach to evaluate B_12_ bioactivity has further uncovered the effect of bacterial innate metabolic capability to convert various B_12_ forms into the catalytically active form. Crude B_12_ extract from *Ectopseudomonas alcaliphila* MSJ19 demonstrated 66% of the enzyme activity and 76% of 3-HP production compared to standard AdoCbl, reinforcing its high bioactive potential. Finally, the extremophilic host was able to produce 3.2 mM 3-HP without external B_12_ supplementation, validating its endogenous B_12_ biosynthetic capability and eliminating the need for complex B_12_ extraction procedures. This positions *Ectopseudomonas alcaliphila* MSJ19 as a promising microbial chassis not only for sustainable B_12_ production but also for broader application in the production of value-added chemicals. Overall, the study offers a scalable, biologically relevant pipeline to assess B_12_ bioactivity across microbial systems and diverse sample sources. Thus, it provides both methodological innovation and foundational insights for metabolic engineering of coenzyme B_12_-dependent pathways.

## Data Availability

The original contributions presented in the study are included in the article/Supplementary material, further inquiries can be directed to the corresponding author.
