# Supplementary material for: Functional characterization of vitamin B12 from an extremophile—Pseudomonas alcaliphila and assessment of its microbial chassis potential
Source: Front Microbiol. 2025 Oct 3;16:1654548. doi: 10.3389/fmicb.2025.1654548 (PMC12531142; doi:10.3389/fmicb.2025.1654548)
Supplement: Supplementary file 1 [file Table_1.docx]

**Supplementary Table 1: Various B_12_ forms supplemented in appropriate concentrations for shake 3-HP production in *Ec*W GD and *Ec*W DD**

| **B_12_ type/form** | **Concentration (nM)** |
| --- | --- |
| *Ectopseudomonas alcaliphila* MSJ19 crude B_12_ extract | 0.35 |
| *Ectopseudomonas alcaliphila* MSJ19 crude B_12_ extract chemically converted to CNCbl form | 0.35 |
| Adenosylcobalamin | 0.35 |
|  | 10 |
|  | 100 |
|  | 500 |
|  | 1000 |
|  | 2000 |
| Methylcobalamin | 0.35 |
|  | 10 |
|  | 100 |
|  | 500 |
|  | 1000 |
|  | 2000 |
| Cyanocobalamin | 0.35 |
|  | 10 |
|  | 100 |
|  | 500 |
|  | 1000 |
|  | 2000 |
| *E. coli* W crude extract (Negative Control) | 0 |

**Supplementary Table 2: Overall summary of binding conformations for molecular docking of various B_12_ ligands with GD and DD respectively**

| **Enzyme** | **Ligands** | **Binding energy (Kcal/mol)** | **Ligand atom** | **Chain** | **Docking_Residues** | | **H-bond distance (A°)** |
| --- | --- | --- | --- | --- | --- | --- | --- |
|  |  |  |  |  | **Donor/Acceptor 1** | **Donor/Acceptor 2** |  |
| **Diol Dehydratase** | Adenosylcobalamin | -7.23 | VAL173 | L | Adenosylcobalamin:D:5AD301:O2′ | Diol_Dehydratase:L:VAL173:O | 2.4 |
|  |  |  | THR172 | L | Adenosylcobalamin:D:5AD301:O2′ | Diol_Dehydratase:L:THR172:OG1 | 2.8 |
|  |  |  | TYR226 | L | Diol_Dehydratase:L:TYR226:HH | Adenosylcobalamin:D:B12300:O3 | 2.2 |
|  |  |  | ASN150 | E | Diol_Dehydratase:E:ASN150:HN | Adenosylcobalamin:D:5AD301:N6 | 2.1 |
|  |  |  | SER301 | L | Diol_Dehydratase:L:SER301:HG | Adenosylcobalamin:D:B12300:O34 | 2.6 |
|  |  |  | GLN156 | E | Diol_Dehydratase:E:GLN156:HE21 | Adenosylcobalamin:D:B12300:O39,N40 | 1.9 |
|  | Cyanocobalamin | -12.9 | GLN156 | E | Diol_Dehydratase:E:GLN156:HE21 | Cyanocobalamin:A:CNC502:O63 | 2.2 |
|  |  |  | ALA157 | E | Diol_Dehydratase:E:ALA157:HN | Cyanocobalamin:A:CNC502:O4, O3 | 2.1 (O4), 1.8 (O3) |
|  |  |  | SER200 | E | Diol_Dehydratase:E:SER200:HG | Cyanocobalamin:A:CNC502:O8R | 1.8 |
|  |  |  | THR172 | L | Diol_Dehydratase:L:VAL173:O | Cyanocobalamin:A:CNC502:N33 | 2.7 |
|  |  |  | VAL173 | L | Diol_Dehydratase:L:THR172:OG1 | Cyanocobalamin:A:CNC502:N29 | 2.6 |
|  | Methylcobalamin | -6.99 | SER224 | L | Methylcobalamin:A:COB300:H292 | Diol_Dehydratase:L:SER224:OG | 2.2 |
|  |  |  | GLN197 | E | Diol_Dehydratase:E:GLN197:HE21 | Methylcobalamin:A:COB300:O5 | 2.2 |
| **Glycerol Dehydratase** | Adenosylcobalamin | -3.93 | SER122 | B | Adenosylcobalamin:D:5AD301:N3 | Glycerol_Dehydrogenase:B:SER122:O | 3.1 |
|  |  |  | THR104 | B | Adenosylcobalamin:D:5AD301:O2′ | Glycerol_Dehydrogenase:B:THR104:OG1 | 3.3 |
|  |  |  | SER225 | A | Glycerol_Dehydrogenase:A:SER225:HG | Adenosylcobalamin:D:B12300:O7R | 2 |
|  |  |  | THR173 | A | Glycerol_Dehydrogenase:A:THR173:HG1 | Adenosylcobalamin:D:B12300:O58 | 1.9 |
|  |  |  | LYS102 | B | Glycerol_Dehydrogenase:B:LYS102:HZ1 | Adenosylcobalamin:D:B12300:O39 | 2.3 |
|  | Cyanocobalamin | -14.5 | SER122 | B | Cyanocobalamin:A:CNC502:O2 | Glycerol_Dehydrogenase:B:SER122:O | 2.8 |
|  |  |  | ASP235 | A | Cyanocobalamin:A:CNC502:N45 | Glycerol_Dehydrogenase:A:ASP235:O | 2.9 |
|  |  |  | ALA124 | B | Glycerol_Dehydrogenase:B:ALA124:HN | Cyanocobalamin:A:CNC502:O4, O3 | 2.1 (O3), 1.8 (O4) |
|  |  |  | THR173 | A | Glycerol_Dehydrogenase:A:THR173:HG1 | Cyanocobalamin:A:CNC502:O28 | 1.6 |
|  | Methylcobalamin | -7.38 | SER122 | B | Methylcobalamin:A:COB300:H622 | Glycerol_Dehydrogenase:B:SER122:O | 1.8 |
|  |  |  | LEU115 | B | Methylcobalamin:A:COB300:H332 | Glycerol_Dehydrogenase:B:LEU115:O | 1.7 |
|  |  |  | SER122 | B | Glycerol_Dehydrogenase:B:SER122:HG | Methylcobalamin:A:COB300:O28 | 2 |
|  |  |  | SER203 | A | Methylcobalamin:A:COB300:H401 | Glycerol_Dehydrogenase:A:SER203:O | 2.2 |
|  |  |  | TYR227 | A | Methylcobalamin:A:COB300:H451 | Glycerol_Dehydrogenase:A:TYR227:OH | 1.7 |
